# Efficacy of abrocitinib in patients with atopic dermatitis resistant to Janus kinase inhibitors

**DOI:** 10.1093/skinhd/vzag045

**Published:** 2026-04-02

**Authors:** Elena Ippoliti, Niccolò Gori, Giulia Coscarella, Flaminia Antonelli, Maria Vittoria Cannizzaro, Ketty Peris

**Affiliations:** Dermatologia, Dipartimento Universitario di Medicina e Chirurgia Traslazionale, Università Cattolica del Sacro Cuore, Rome, Italy; Dermatologia, Dipartimento di Scienze Mediche e Chirurgiche, Fondazione Policlinico Universitario A. Gemelli IRCCS, Rome, Italy; Dermatologia, Dipartimento Universitario di Medicina e Chirurgia Traslazionale, Università Cattolica del Sacro Cuore, Rome, Italy; Dermatologia, Dipartimento di Scienze Mediche e Chirurgiche, Fondazione Policlinico Universitario A. Gemelli IRCCS, Rome, Italy; Dermatologia, Dipartimento Universitario di Medicina e Chirurgia Traslazionale, Università Cattolica del Sacro Cuore, Rome, Italy; Dermatologia, Dipartimento di Scienze Mediche e Chirurgiche, Fondazione Policlinico Universitario A. Gemelli IRCCS, Rome, Italy; Dermatologia, Dipartimento Universitario di Medicina e Chirurgia Traslazionale, Università Cattolica del Sacro Cuore, Rome, Italy; Dermatologia, Dipartimento di Scienze Mediche e Chirurgiche, Fondazione Policlinico Universitario A. Gemelli IRCCS, Rome, Italy; Dermatologia, Dipartimento Universitario di Medicina e Chirurgia Traslazionale, Università Cattolica del Sacro Cuore, Rome, Italy; Dermatologia, Dipartimento di Scienze Mediche e Chirurgiche, Fondazione Policlinico Universitario A. Gemelli IRCCS, Rome, Italy; Dermatologia, Dipartimento Universitario di Medicina e Chirurgia Traslazionale, Università Cattolica del Sacro Cuore, Rome, Italy; Dermatologia, Dipartimento di Scienze Mediche e Chirurgiche, Fondazione Policlinico Universitario A. Gemelli IRCCS, Rome, Italy

## Abstract

Abrocitinib, a selective Janus kinase 1 (JAK1) inhibitor, has demonstrated efficacy in moderate-to-severe atopic dermatitis (AD), although data in patients whose AD is resistant to multiple systemic therapies remain limited. We report the case of six patients with moderate-to-severe AD who were unresponsive to previous treatments, including dupilumab, tralokinumab, lebrikizumab, upadacitinib and/or baricitinib. All patients received abrocitinib 200 mg daily. After only 16 weeks, five patients achieved a good clinical response, defined as an Eczema Area Severity Index score of ≤7 and a reduction of ≥3 points in the itch Numerical Rating Scale; in three patients, this response was maintained through 32 weeks. Two patients discontinued abrocitinib due to primary or secondary inefficacy. No serious adverse events were reported; only one patient experienced mild increases in cholesterol and triglyceride levels. These findings suggest that patients with difficult-to-treat AD may benefit from therapy with abrocitinib, potentially due to its high selectivity for JAK1, which may explain its efficacy where other JAK inhibitors have failed.

## Teaser

Abrocitinib, a selective JAK1 inhibitor, showed efficacy in patients with severe atopic dermatitis unresponsive to multiple systemic therapies. We report the case of six patients; five out of six achieved a good clinical response by week 16, with a high safety profile.

What is already known about this topic?

- Atopic dermatitis (AD) is a chronic inflammatory skin disease that has a significant impact on patients’ quality of life.
- Long-term treatment of moderate-to-severe AD involves biologics targeting interleukin (IL)-4/IL-13 or oral Janus kinase (JAK) inhibitors.

What does this study add?

- We provide preliminary evidence regarding the potential benefit of abrocitinib in patients with AD that is resistant to other biologics or JAK inhibitors.
- Abrocitinib’s higher JAK1 selectivity may explain its efficacy where other JAK inhibitors have failed.

Atopic dermatitis (AD) is the most common chronic inflammatory skin disease worldwide, and has a significant impact on patients’ quality of life, particularly in its moderate-to-severe forms.^1^ Currently, the long-term management of moderate-to-severe AD is achieved through two main approaches: targeting the interleukin (IL)-4/IL-13 axis with biologics as dupilumab, tralokinumab and lebrikizumab, and targeting the Janus kinase (JAK) enzymes pathways using selective oral JAK inhibitors, including baricitinib, abrocitinib and upadacitinib.^2^ The JAK family, comprising JAK1, JAK2, JAK3 and tyrosine kinase 2 (TYK2), plays a crucial role in AD pathogenesis by mediating the signalling of type II cytokine receptors and modulating the effects of several cytokines such as IL-4, IL-13, IL-22 and IL-31.^3^ Abrocitinib, a selective inhibitor of the JAK1 isoform, showed high efficacy in the treatment of moderate-to-severe AD, with over 50% of patients achieving Eczema Area Severity Index (EASI) 90, defined as an improvement ≥90% of baseline EASI, in randomized clinical trials at a dosage of 200 mg daily.^4^ However, data on the efficacy of abrocitinib in patients with AD resistant to multiple treatments, including biologics and JAK inhibitors, are lacking. We analysed the efficacy of abrocitinib in six patients with moderate-to-severe AD, the majority of whom were resistant to previous treatments with dupilumab, tralokinumab, lebrikizumab, upadacitinib and/or baricitinib.

## Case report

**Figure 1:**
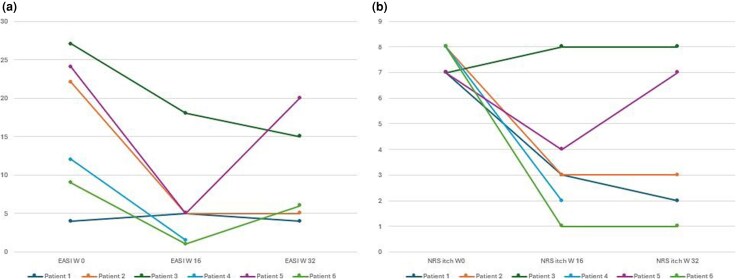
(a) Eczema Area Severity Index (EASI) score values at 0, 16 and 32 weeks (W). For patient 4, follow-up data are available only up to week 16. (b) Itch Numerical Rating Scale (NRS) score values at 0, 16 and 32 weeks. For patient 4, follow-up data are available only up to week 16.

**Figure 2:**
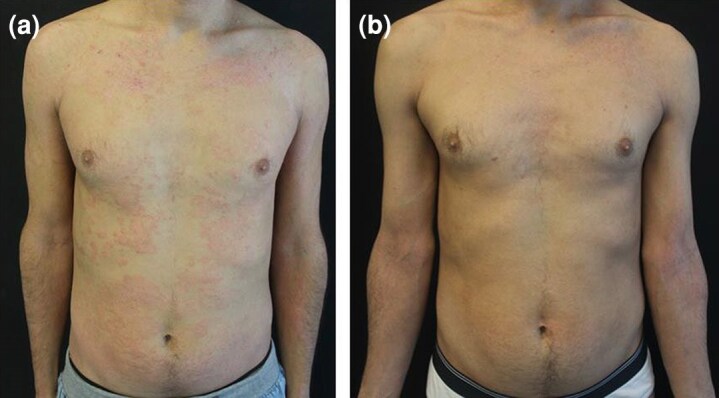
(a) Widespread atopic dermatitis lesions on the trunk in a patient resistant to multiple therapies at baseline. (b) Improvement of lesions at week 16 of treatment with abrocitinib 200 mg daily.

**Figure 3:**
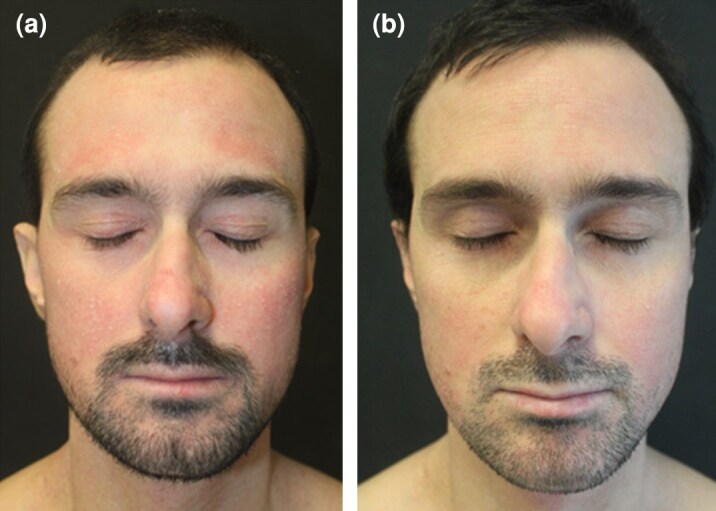
(a) Eczematous lesions located on the head at baseline. (b) Eczematous lesions located on the head after 16 weeks of therapy with abrocitinib 200 mg daily.

Six patients, including two women and four men [mean (SD) age 34 (9) years], with moderate-to-severe AD resistant to previous biologics or JAK inhibitors were then treated with abrocitinib 200 mg. All six patients had AD since childhood; three patients had asthma (*n* = 1), allergic rhinitis (*n* = 1) and rhinoconjunctivitis associated with keratoconus (*n* = 1). Past treatments included topical corticosteroids and systemic ciclosporin for all patients. In addition, dupilumab was administered to all of six patients but discontinued due to primary inefficacy (two patients), loss of efficacy (three patients) and development of a diffuse drug-related maculopapular itchy eruption 2 months after treatment initiation (one patient). All six patients had also been treated previously with upadacitinib, at a daily dose of 30 mg, which was discontinued due to loss of efficacy in five patients, while one patient experienced primary inefficacy. Two patients who did not respond to therapy with upadacitinib were subsequently treated with baricitinib, which was discontinued after 3 months due to inefficacy in both cases. One patient discontinued therapy with tralokinumab due to inefficacy and one with lebrikizumab due to loss of efficacy. The details regarding previous treatments are specified in Table 1. All six patients showed a classic phenotype, severely affecting the folds, head and neck area, with involvement of the hands in two patients. Mean (SD) EASI score at baseline was 16.3 (8.5) and mean (SD) Itch Numerical Rating Scale (NRS) at baseline was 7.5 (0.5). Given the difficulty in treating this cohort of patients, abrocitinib was prescribed at a daily dosage of 200 mg. A significant improvement in clinical signs and symptoms, assessed by reductions in EASI and Itch NRS scores, was observed in the majority of patients after 16 and 32 weeks of treatment (Figures 1–3). For patient 4, follow-up data were only available up to week 16.

**Table 1:** Patients’ therapy timelines showing treatment periods, administered drugs and reasons for discontinuation

Patient	Period	Therapy	Reason for discontinuation
1	January 2020 (10 days), August–December 2023	Ciclosporin 150 mg daily, upadacitinib 30 mg daily	Discontinued (relapse), discontinued (inefficacy)
	February–April 2024	Dupilumab 600 mg at baseline followed by 300 mg every 2 weeks	Discontinued (macular papular itchy eruption)
	March 2024–present	Abrocitinib 200 mg daily	Ongoing
2	January–June 2020	Ciclosporin 200 mg daily	Discontinued (inefficacy)
	November 2022–March 2023	Upadacitinib 30 mg daily	Discontinued (loss of efficacy)
	April–September 2023	Dupilumab 600 mg at baseline followed by 300 mg every 2 weeks	Discontinued (inefficacy)
	September 2023–present	Abrocitinib 200 mg daily	Ongoing
3	Febuary–April 2019	Ciclosporin 250 mg daily	Discontinued (disease remission)
	May–November 2021	Dupilumab 600 mg at baseline followed by 300 mg every 2 weeks	Discontinued (inefficacy)
	November 2021–May 2023	Upadacitinib 30 mg daily	Discontinued (loss of efficacy)
	May–July 2023	Tralokinumab 600 mg at baseline followed by 300 mg every 2 weeks	Discontinued (inefficacy)
	July 2023–present	Abrocitinib 200 mg daily	Ongoing
4	October 2019–November 2022	Dupilumab 600 mg at baseline followed by 300 mg every 2 weeks	Discontinued (loss of efficacy)
	September–October 2022	Ciclosporin 200 mg daily	Discontinued (inefficacy)
	November 2022–July 2023	Upadacitinib 30 mg daily	Discontinued (loss of efficacy)
	July–October 2023	Baricitinib 4 mg daily	Discontinued (inefficacy)
	June–December 2024	Lebrikizumab 500 mg at baseline, at week 2, followed by 250 mg every 2 weeks until week 16, then 250 mg every month	Discontinued (loss of efficacy)
	February 2025–present	Abrocitinib 200 mg daily	Ongoing
5	April–July 2019	Ciclosporin 250 mg daily	Discontinued (inefficacy)
	July 2019–June 2020	Dupilumab 600 mg at baseline followed by 300 mg every 2 weeks	Discontinued (loss of efficacy and conjunctivitis)
	May 2023–April 2024	Upadacitinib 30 mg daily	Discontinued (loss of efficacy)
	June 2024–present	Abrocitinib 200 mg daily	Ongoing
6	April–June 2021	Dupilumab 600 mg at baseline followed by 300 mg every 2 weeks	Discontinued (loss of efficacy)
	Auguest 2022–January 2024	Upadacitinib 30 mg daily	Discontinued (loss of efficacy)
	Febuary–May 2024	Baricitinib 4 mg daily	Discontinued (inefficacy)
	May 2024, 2 weeks	Ciclosporin 200 mg daily	Discontinued (disease flare))
	May 2024–present	Abrocitinib 200 mg daily	Ongoing

Endpoints considered to represent an adequate clinical response, namely an absolute EASI score ≤7 and a reduction of at least 3 points on the itch NRS,^5^ were achieved at 16 weeks by five of six patients. Two of six patients discontinued abrocitinib due to primary and secondary inefficacy, respectively. No serious adverse events were observed. One patient exhibited a mild elevation of cholesterol (228 mg dL^–1^; normal <200) and triglycerides (184 mg dL^–1^; normal <150).

## Discussion

In recent years, the advent of new systemic therapies targeting the key pathogenic mechanisms of AD has revolutionized disease management, enabling long-term control of its clinical signs and symptoms.^1^ Nevertheless, a proportion of patients demonstrate either an inadequate response or a secondary loss of efficacy to one or more systemic therapies. In our small case series, abrocitinib was associated with short-term clinical benefit in patients with difficult-to-treat AD in whom multiple systemic therapies, including biologics and JAK inhibitors, had failed to treat the AD, with five of six patients achieving an adequate response (EASI ≤7) after 16 weeks of treatment. Good clinical control, defined by an EASI score ≤7 and a reduction of at least 3 points on the itch NRS,^5^ was then maintained throughout the 32 weeks of observation in three of the six patients. Notably, AD in all six patients was either resistant or intolerant to dupilumab. In this regard, the JADE DARE trial demonstrated that patients whose AD was resistant to dupilumab could benefit from abrocitinib at a daily dose of 200 mg,^6^ a finding also supported by real-world observations.^7^ In addition, our patients exhibited either primary or ­secondary inefficacy to upadacitinib, with two patients also showing a poor response to baricitinib. A recent network meta-analysis comparing binary efficacy outcomes of systemic treatments for AD identified upadacitinib 30 mg daily and abrocitinib 200 mg daily as the most ­effective treatments for moderate-to-severe AD.^8^ Both upadacitinib and abrocitinib selectively target JAK1; however, the selectivity of abrocitinib differs slightly, as it has a higher selectivity for JAK1 (half-maximal inhibitory concentration, IC_50_: 29 nmol) compared with upadacitinib (IC_50_: 45 nmol). Additionally, its activity towards JAK2 and JAK3 is markedly lower than that of upadacitinib.^9^ These pharmacodynamic differences might partially explain the observed efficacy of abrocitinib in our patients previously treated unsuccessfully with upadacitinib; however, this observation should be interpreted with caution due to the limited sample size. Regarding safety, no major adverse events were reported in our case series. A mild increase in cholesterol and triglyceride levels was observed in one patient, consistent with previous reports of an approximate 10% increase in plasma lipoprotein levels.^10^

Our case series highlights the short-term potential benefit of abrocitinib in patients with difficult-to-treat AD that has not successfully been treated with previously with biologics and other JAK inhibitors. Given the small sample size and the absence of a control group, additional data from larger, controlled studies are needed to corroborate these preliminary findings.
